# Supplementary material for: The Evolution of the Satratoxin and Atranone Gene Clusters of Stachybotrys chartarum
Source: J Fungi (Basel). 2022 Mar 24;8(4):340. doi: 10.3390/jof8040340 (PMC9027890; doi:10.3390/jof8040340)
Supplement: Supplementary file 1 [file jof-08-00340-s001.zip › jof-1621592-sm-2nd conversion.pdf]

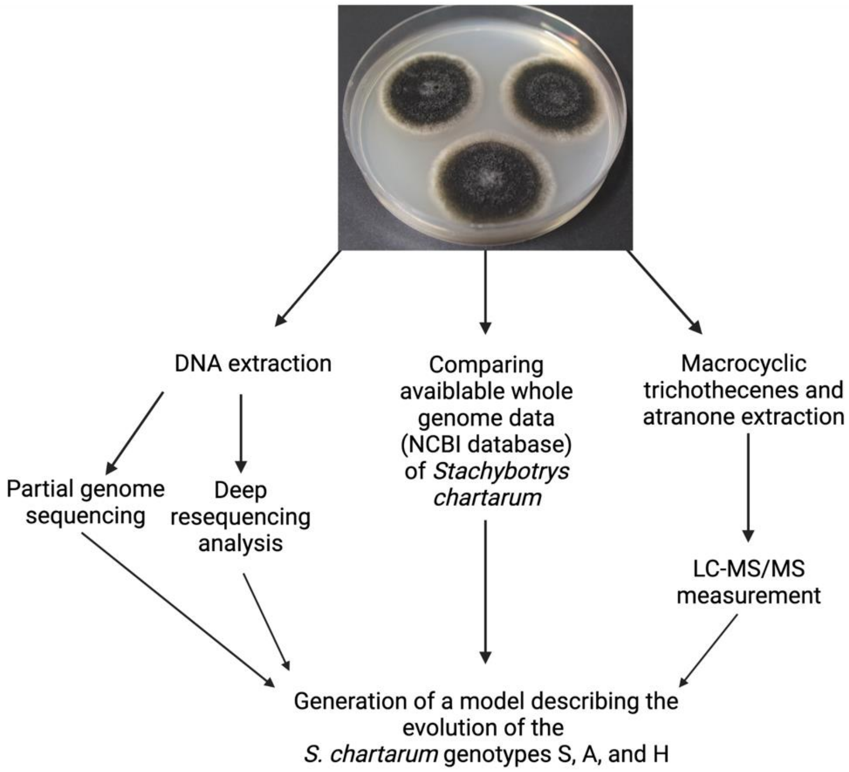

Figure S1. Schematic diagram of the performed work during this study.

(A)

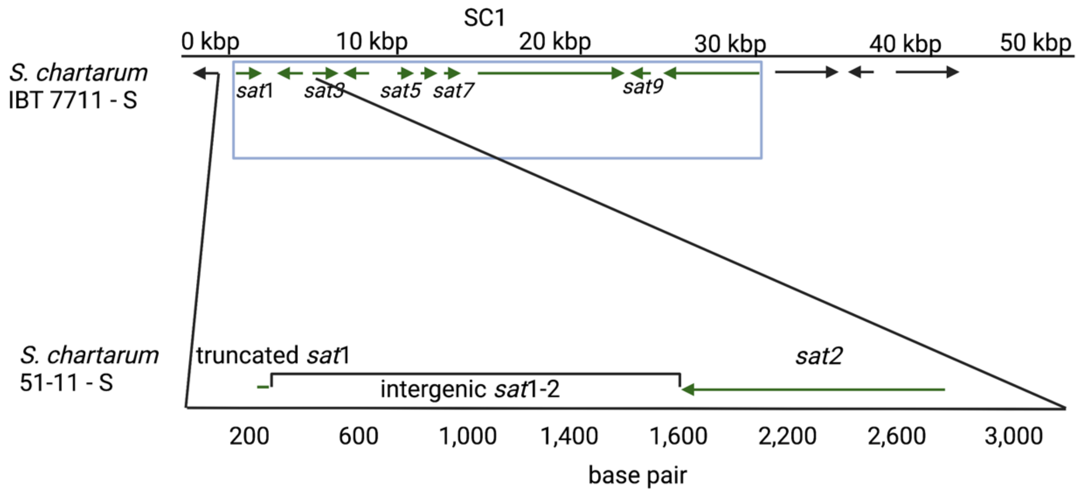

(B)

IBT7711 AGCAAACCGCGCGAGCAAATGCATTATACTGCGCTACGCGCGAACCCTACGGCTGACTGG 60

51-11 -- 0

IBT7711 AATCGGGAGTTTCCATTCACAGCCTGTTATTGAACAATGTGGGCCAGTTTCCCTAGACCA 120

```
51-11          ------------------------------------------------------------
        0

IBT7711        GAAGCCATTCCGCCGGGATACGTCGGCGAAACCCAGCACCAACATCGCTCCATCATGCTT
180
51-11          ------------------------------------------------------------
        0

IBT7711        CTTAATGGCAAATCCCGCAGTTGGATCTTCTTGTACGAGAGGCTGCCCGCACCAAGCCAC
240
51-11          ------------------------------------------------------------
        0

IBT7711        GACCGCGTAAAGTGCATTGCTGAAGATGTCATCGAATTCGCTGATAGCTTCGCGGATTGG
300
51-11          ------------------------------------------------------------
        0

IBT7711        AGTATTTGGAACAATACAAAGCTTGAAGACGTGGTCGACCATTCGACTGCAGGTATGTCT
360
51-11          ------------------------------------------------------------
        0

IBT7711        AACTTGGAAGAAGGAATCGTCAAGAACTTCAGTCATGGCCGCATTGTCCTTGTCGGCGAT
420
51-11          ------------------------------------------------------------
        0

IBT7711        GCTTGCCATAAATTTACCTCTAACGCTGGCCTTGGCTTGAATAATGGTATCCAGGATATT
480
51-11          ------------------------------------------------------------
        0

IBT7711        GTTGCAGGCTGCAACAGCATTCGCAAGGTCGTCACAGAGTCCGGGTTTGATCTGCCGGAT
540
51-11          ------------------------------------------------------------
        0
```

```
IBT7711      GTCAAAGCATTAGAAGCGACTTTCAAAACATACTATGAAATGAGGCTGGGACCTTTCAAC
600
51-11        ------------------------------------------------------------
        0


IBT7711      GACGATTTCATTCATTCAAAAATGATGACCCGCATGCAAGCATGGGCAAATACCTGGTAT
660
51-11        ------------------------------------------------------------
        0


IBT7711      TTTCTCTTTACCAGATATCTTTTCTTCATCTTCTCAGAGTGGATCTTGTTCCGCTTTACC
720
51-11        ----------------TAGAATTCTTCAGCTTTT-----------------------AAT
21
                                  ******* *** *                       *

IBT7711      ATGTTGCGAAGGGTCTGTACCGGTTTGGTTTTGACTATGCACCTGGCAAAGAGCCGTTTG
780
51-11        ATAATTTAA--------TTATGCTTTCTTAT---TCTAGCTCCTTAATTATAACCAATTA
70
             **  *   *        *   * ***  * *       ** ***     * * **  **

IBT7711      GTGGCACTTTTGAATGGGTTTATCCGATCAGGGTATCACTCATTCATCTGGATCAGCGCA
840
51-11        ATAATATAGTACCTAGTATATCCCTAACCT----------GCTTAAGATAAAGCCTTATA
120
              *   *   *     *  * *  *  * *             ** *  *  * *     *

IBT7711      ATTCGACCTTGCAATGATCAG---CTGTTGAACCAACTCTTAGCT-GGTGCGATTCAAAT
896
51-11        CTTAAACCTGAATATAAACCCTTGAGGTTGAACTTCATCTTTGAAGGAATATCCTACTCT
180
              **  ****    ** * *       *******    **** *   *       *    *

IBT7711      CGAAATTCTTTGCTCCCTGAATACTTGGCGAATTTCTTCATGTCGTGCGCCATTACTCTT
956
51-11        TGACCTAACCCGTTGCCCTAATCCGGCAAGACCTGGTCAGAGACATTACACATTACTCTT
240
              **  *     * * **  *** *     **  *  *    * * *    **********
```

IBT7711

GGTGTTCGATCAAGCAAGGGGCTAGGTATAGGGTGGCATGCGGGATGGACAAGGTTGAAG1016

51-11 GGTGTTTGATCAAGCAAGGGGCTAGGTATAGGGTGGCATGCGGGATGGACAAGGTTGAAG

300

****** ***

IBT7711

CGCAGTCCACACTTTGGAATAAGCTTCCCTAAACCAAGTGTGATTCAAAACCCAGTGACG1076

51-11

CGCAGTCCACACTTTGGAATAAGCTTCCCTAAACCAAGTGTGATTCAAAACCCAGTGACG360

****

IBT7711

GAACCCCAATGATAGTGCCAGATACATCCTTTCTAGGCGACGCCGAAGCCGTCTTCAATC1136

51-11 GAACCCCAATGATAGTGCCAGATACATCCTTTCTAGGCGACGCCGAAGCCGTCTTCAATC

420

****

IBT7711

TTCTTCTTGGTCGTATTACAAGAAGACTCTGGACCTAGCAATTTGTCCATTGGCCAAAGA1196

51-11 TTCTTCTTGGTCGTATTACAAGAAGACTCTGGACCTAGCAATTTGTCCATTGGCCAAAGA

480

****

IBT7711

TAGGCTCCGTTGCAGGATCCATCATCAGATCGAAGAAGAGTGATTGAAAATCAACTTCCC1256

51-11 TAGGCTCCGTTGCAGGATCCATCATCAGATCGAAGAAGAGTGATTGAAAATCAACTTCCC

540

****

IBT7711

TGTCTCGCAGAAGCTGATCAATCAGATGCCTTACACATTTTTTTAAATTGGTTGAGGTTC1316

51-11 TGTCTCGCAGAAGCTGATCAATCAGATGCCTTACACATTTTTTTAAATTGGTTGAGGTTC

600

****

IBT7711

TGTACTGCGAGCGCGTGACCAGATGGAGCAGGAGTGAAGGAACACAAATTGTTCTAATAT1376

51-11 TGTACTGCGAGCGCGTGACCAGATGGAGCAGGAGTGAAGGAACACAAATTGTTCTAATAT

660

****

IBT7711

CTTGTTCGAAATGGAAACGAGGCGGGCTTTCTACAAAGACCGATAGCTTGCAGAATACGC1436

51-11 CTTGTTCGAAATGGAAACGAGGCGGGCTTTCTACCAAGACCGATAGCTTGCAGAATACGC
720
********************************** *************************

IBT7711
AACACCAGATCCACGTCAGGCGAACGGTTGACATGGCACTTGAAGCAACGGACTCATGAT1496
51-11 AACACCAGATCCACGTCAGGCGAACGGTTGACATGGCACTTGAAGCAACGGACTCATGAT
780
****

IBT7711
AATTAGCAGCTGGCTGGTACGGGTCTGATACATCTTGAACATTCCTTGGTATATGCAGTC1556
51-11 AATTAGCAGCTGGCTGGTACGGGTCTGATACATCTTGAACATTCCTTGGTATATGCAGTC
840
****

IBT7711
GGTGAAAAGAAAAATATCGGGCGCCGGTGGAGATTTGCAATTCGTATTGAGCACCACTAT1616
51-11 GGTGAAA-GAAAAATATCGGGCGCCGGTGGAGATTTGCAATTCGTATTGAGCACCACTAT
899
******* **

IBT7711
TGAGAGAGTCACCTCTGACAGTGAATGAATCACTCTTCCTGGCCTAGCAGTGTTGTCCTT1676
51-11 TGAGAGAGTCACCTCTGACAGTGAATGAATCACTCTTCCTGGCCTAGCAGTGTTGTCCTT
959
****

IBT7711
CCATTCTTGGGTATGCAGGGCTAATACTCATTCTATCTTATCGTTTGGATCACATAAGCG1736
51-11
CCATTCTTGGGTATGCAGGGCTAATACTCATTCTATCTTATCGTTTGGATCACATAAGCG1019
****

IBT7711
GCATACAGTTCATCTATTTTCTGGATTGAGGCTTATCCTCGTATCCTTGCATGGCCATGG1796
51-11
GCATACAGTTCATCTACTTTCTGGATTGAGGCTTATCCTCGTATCCTTGCATGGCCATGG1079
**************** ***

IBT7711 TAATCTTTTACCTTCCTGAGCTGGTCCCAAAGTTGCACAGCGGGCGG-
GACTTTTGTTTT1855
51-11
TAATCTTTTACCTTCCTGAGCTGGTCCCAAAGTTGCACAGCGGGCGGGGACTTTTGTTTT1139

```
            ******************************************** ***********

IBT7711
GCCTAGCCGTAGGAGGGTAGTATCTGACCTTATATGCAGCTAGAGTGGTGGGCTCGGACA1915
51-11
GCCTAGCCGTAGGAGGGTAGTATCTGACCTTATATGCAGCTAGAGTGGTGGGCTCGGACA1199
            ********************************************************

IBT7711
TAGGCCAATCACATCGCTACGCGTCGGTTGTCTTTTATTCCGGCCGCAATAGGAAAAAAG1975
51-11
TAGGCCAATCACATCGCTACGCGTCGGTTGTCTTTTATTCCGGCCGCAATAGGAAAAAAG1259
            ********************************************************

IBT7711
CGAAGAGCATATCTGTTCTGACTCCGACTAAATCCCAATTTTATGCAAGGGATACAATCA2035
51-11
CGAAGAGCATATCTGTTCTGACTCCGACTAAATCCCAATTTTATGCAAGGGATACAATCA1319
            ********************************************************

IBT7711
GCATAATGTAACCCCAACCACCCCTTGTCGAGTCGTCTCACAGCCTGCGCCCGTCCGCGC2095
51-11
GCATAATGTAACCCCAACCACCCCTTGTCGAGTTGTCTCACAGCCTGCGCCCGTCCGCGC1379
            ****************************** *************************

IBT7711
GCATTGCAGCCAACATCAGGCGCCAGCCCCCCTAAATCCCTTCATCTGGCCATACCTCCG2155
51-11
GCATTGCAGCCAACATCAGGCGCCAGCCCCCCTAAATCCCTTCATCTGGCCATACCTCCG1439
            ********************************************************

IBT7711
ATATCGCAAGTGCTTGCCTCTCGGACATCTTTGCCATCATATGCACCACAGGCAGGAGGC2215
51-11
ATATCGCAAGTGCTTGCCTCTCGGACATCTTTGCCATCATATGCACCACAGGCAGGAGGC1499
            ********************************************************

IBT7711
ACGGAGTAGACTGGATCAATATTATTGGAATTGCAATGTAGAGGCTACTTGATGGGTGAT2275
51-11
ACGGAGTAGACTGGATCAATATTATTGGAATTGCAATGTAGAGGCTACTTGATGGGTGAT1559
            ********************************************************
```

IBT7711

GCCTTTGCTGAGTTCTGGTGAAGGCAGAGGATTACTCATTCGATTTGAACGAAAGATCTA2335

51-11

GCCTTTGCTGAGTTCTGGTGAAGGCAGAGGATTACTCATTCGATTTGAACGAAAGATCTA1619

****

IBT7711

GACGATCCACGAAAACAACCTAGGCGATCGTCGCATTCTTCTTCGCGATGTCATCTAGTG2395

51-11

GACGATCCACGAAAACAACCTAGGCGATCGTCGCATTCTTCTTCGCGATGTCATCTAGTG1679

****

IBT7711

TCCTGATAGTATGGTCCCAGGCCTTCTTGCTAGTATCAGGTTTGCGCGACTTCTCGAACA2455

51-11

TCCTGATAGTATGGTCCCAGGCCTTCTTGCTAGTATCAGGTTTGCGCGACTTCTCGAACA1739

****

IBT7711

TGATCTGCGGTGTCTGCGAATCTGCCCTTTGACCGAGACTGTATGCTCCGCCACCGACTA2515

51-11

TGATCTGCGGTGTCTGCGAATCTGCCCTTTGACCGAGACTGTATGCTCCGCCACCGACTA1799

****

IBT7711

CTCCCAACGAGCTCTTGGGTGCCAGCGTGTCCGTCGACGTCGGAGCATCTTGGCCAGACC2575

51-11

CTCCCAACGAGCTCTTGGGTGCCAGCGTGTCCGTCGACGTCGGAGCATCTTGGCCAGACC1859

****

IBT7711

CCTTTGCCGAAAACCTGGAAGTGGCTAGATAAGCCATCACCATGCCGCAGTCTTCGGAAC2635

51-11

CCTTTGCCGAAAACCTGGAAGTGGCTAGATAAGCCATCACCATGCCGCAGTCTTCGGAAC1919

****

IBT7711

GTGTCATGTACAACGACGTCAAAGGCTTCATAAGCGTGAACAACACTCTGAACCAGCGAG2695

51-11

GTGTCATGTACAACGACGTCAAAGGCTTCATAAGCGTGAACAACAGTCTGAACCAGCGAG1979

*** **************

IBT7711

GCATCTGTGTAAACCCCGGTCCATCGACCAGCCCTGGATAAATGTGAATCAAGCTGAGGC2755

51-11

GCATCTGTGTAAACCCCGGTCCATCGACCAGCCCTGGATAAATGTGAATCAAGCTGAGGC2039

****

IBT7711

GTTCAGCGTACTTCTCTGCCAACTCCTCGAAAACAAAGGTTTTCATGAAGGTTGTGTATT2815

51-11

GTTCAGCGTACTTCTCTGCCAACTCCTCGAAAACAAAGGTTTTCATGAAGGTTGTGTATT2099

****

IBT7711

TGCGGACGGTGTTGAAGTGGTAGATTTCTGCCGGAACAAAGCCAATGGGCTCTTCGTCTG2875

51-11

TGCGGACGGTGTTGAAGTGGTAGATTTCTGCCGGAACAAAGCCAATGGGCTCTTCGTCTG2159

****

IBT7711

GTTTGACACCGTTCTCCATCCCGCCAGCATAGACAGAAATGACATGGGCTACGCGAGGCG2935

51-11

GTTTGACACCGTTCTCCATCCCGCCAGCATAGACAGAAATGACATGGGCTACGCGAGGCG2219

****

IBT7711

AAGCAGTAAGGAGTGGCAAGAGCTGCAGGATGAACCGGATCCGCGAGTAGTAGATTGTGG2995

51-11

AAGCAGTAAGGAGTGGCAAGAGCTGCAGGATGAACCGGATCCGCGAGTAGTAGATTGTGG2279

****

IBT7711

ATTCCAAGGCATCAAGACCCTCTTCGGTAGCTAAGGCATCGCGGTCAGTCCAATGTGCCA3055

51-11

ATTCCAAGGCATCAAGACCCTCTTCGGTAGCTAAGGCATCGCGGTCAGTCCAATGTGCCA2339

****

IBT7711

GTTCAACGCGAAGCAATCTTGGAACCCGAATACTCACTGCGCTTATGGCCGAGGATAGGA3115

51-11

GTTCAACGCGAAGCAATCTTGGAACCCGAATACTCACTGCGCTTATGGCCGAGGATAGGA2399

****

IBT7711

ATGGCGTGCGTCATGTACAGGAGATCGAGACGAGCCAGCTCTCCGTGAAATGGAGCCTCG3175

51-11

ATGGCGTGCGTCATGTACAGGAGATCGAGACGAGCCAGCTCTCCGTGAAATGGAGCCTCG2459

```
              ************************************************************

IBT7711
GTCTCTTGCTTGATAATTTCTGCGCTGGACTTATCAACCTCGCTGATGAGCGCAAGGTCT3235
51-11
GTCTCTTGCTTGATAATTTCTGCGCTGGACTTATCAACCTCGCTGATGAGCGCAAGGTCT2519
              ************************************************************

IBT7711
GTCGCGTGGATAAAACGCCAGTCTGACCCTGGGCTGATCTTCTGGCACTCTGAAATGACT3295
51-11
GTCGCGTGGATAAAACGCCAGTCTGACCCTGGGCTGATCTTCTGGCACTCTGAAATGACT2579
              ************************************************************

IBT7711
GTCTTGGCGCGTCCGGCATTCCGCCCGACGATGTACACGCGGAGCTTGCTGCCATGGCTG3355
51-11
GTCTTGGCGCGTCCGGCATTCCGCCCGACGATGTACACGCGGAGCTTGCTGCCATGGCTG2639
              ************************************************************

IBT7711
GCAAAAGTCGTGGCCAAGGCTTTGGCTAGGTACGAGCCAATGCCCGTGGTGCCGCCAGCG3415
51-11
GCAAAAGTCGTGGCCAAGGCTTTGGCTAGGTACGAGCCAATGCCCGTGGTGCCGCCAGCG2699
              ************************************************************

IBT7711
ACGGCTGCGACAATGGACGATCCCTGGGGCAGTTCCGCCACTGCAGCCCGAATGACCTGA3475
51-11
ACGGCTGCGACAATGGGCGATCCCTGGGGCAGTTCCGCCACTGCAGCCCGAATGACCTGA2759
              **************** *******************************************

IBT7711
AGGCTAGGCATTATCGGCTTCAGGTACAGCGAAAGTGAAGCTCGGCAGCTGACTCAAGAT3535
51-11
AGGCTAGGCATTATCGGCTTCAGGTACAGCGAAACTGAAGCTCGGCAGCTGACTCAAGAT2819
              ********************************** *************************

IBT7711
GGTACAGGCGACCTAGTTTGAAATCGAAGGTAGATGTGAACGGCTTGGCCACAGCGCTGG3595
51-11
GGTACAGGCGACCTAGTTTGAAATCGAAGGTAGGTGTGAACGGCTTGGCCACAGCGCTGG2879
              ********************************* **************************
```

IBT7711

AAGGATTTATACGGAAGATGACAGTAGCATTGATGGGGTAGCCGTAAATATGGGTTCAAT3655

51-11

AAGGATTTATACGGAAGATGACAGTAGCATTGATGGGGTAGCCGTAAATATGGGTTCAAT2939

****

IBT7711

GCCAGGGATGATGTTCTAAGCTAGGCTGCGGGCGCTAAACCGTAGTATAAATTAAAATTA3715

51-11

GCCAGGGATGATGTTCTAAGCTAGGCTGCGGGCGCTAAACCGTAGTATAAATTAAAATTA2999

****

IBT7711

AGGGAGCTGCTTATATAATAGTGGGACTCTTTAAGAAACGATATTTAGCTTATATGAATC3775

51-11

AGGGAGCTGCTTATATAATAGTGGGACTCTTTAAGAAACGATATTTAGCTTATATGAATC3059

****

IBT7711

TAAAGGACGATTTCTTAAAACTATTATATTATATTCTTAGGATTATAAGCTTACTTTTTA3835

51-11 TAAAGGACGATTTCTTAAAACTATTATATTATATTCTTAGGATTATAAGCTTACTT--

TA3117

** **

IBT7711

ATTAAATTTTTGATAAAATAACCAAATATTCTTCCCACTTTATTTTTATTCTTTAAAAGT3895

51-11

ATTAAATTTTTGATAAAATAACCAAATATTCTTCCCACTTTATTTTTATTCTTTAAAAGT3177

****

IBT7711

AATTGCTCATCGCGACCCTCACCACGACATCTATGTCTAAGATCATGGATACTGCAAGCT3955

51-11

AATTGCTCATCGCGACCCTCACCACGACATCTATGTCTAAGATCATGGATACTGCAAGCT3237

****

IBT7711

TATTCCGAGTAGATGGCATGGTGGCCGCGGTGACAGGTGGAGGAACAGGCAAGGCTTACC4015

51-11

TATTCCGAGTAGATGGCATGGTGGCCGCGGTGACAGGTGGAGGAACAGGCAAGGCTTACC3297

****

IBT7711 ACCTTCACATTAAGAGCATAACTAATATGCAGCATCGTAGGAATCGGTCTGATG 4069

51-11 ACCTTCACATTAAGAGCATAACTAATATGCAGCATCGTAGGAATCGGTCTGATG 3351

* *

Figure S2. (A) Truncated *sat*1 in scaffold 31 of 51-11 annotated according to Semeiks *et al*. (2014); (B) Alignment of the annotated area of IBT 7711 and 51-11 shown in panel A; *sat*1 and *sat*2 of IBT 7711 colored in green; * indicates positions which have a single, fully conserved residue.

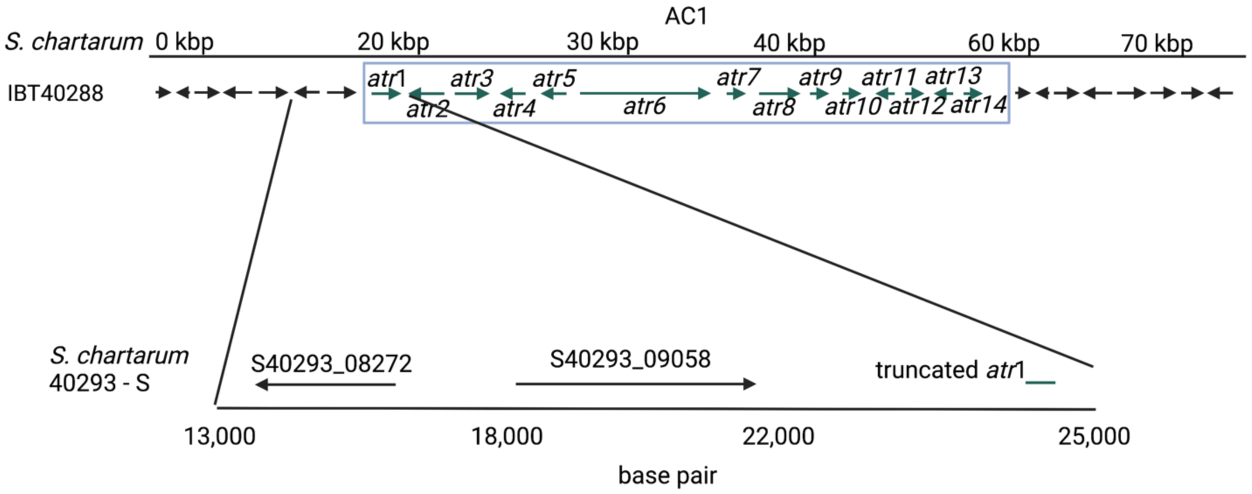

Figure S3. Truncated *atr*1 in scaffold 1474 of IBT 40293 and two genes encoding hypothetical proteins, both found downstream of the AC1 in A-type strain IBT 40288; Arrows depict genes. Green arrows are part of the gene cluster.

(A)

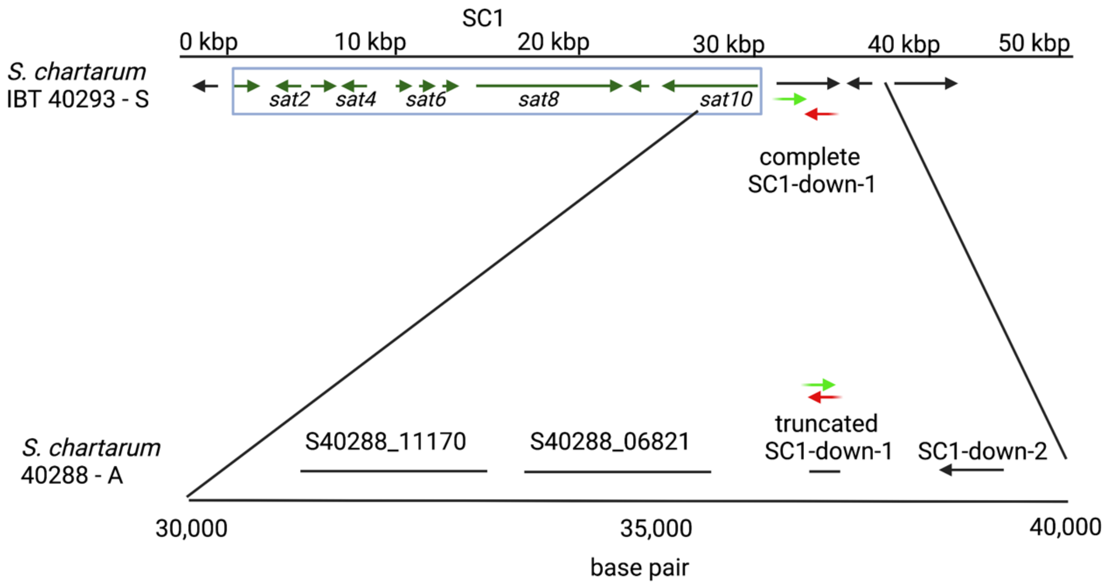

(B)

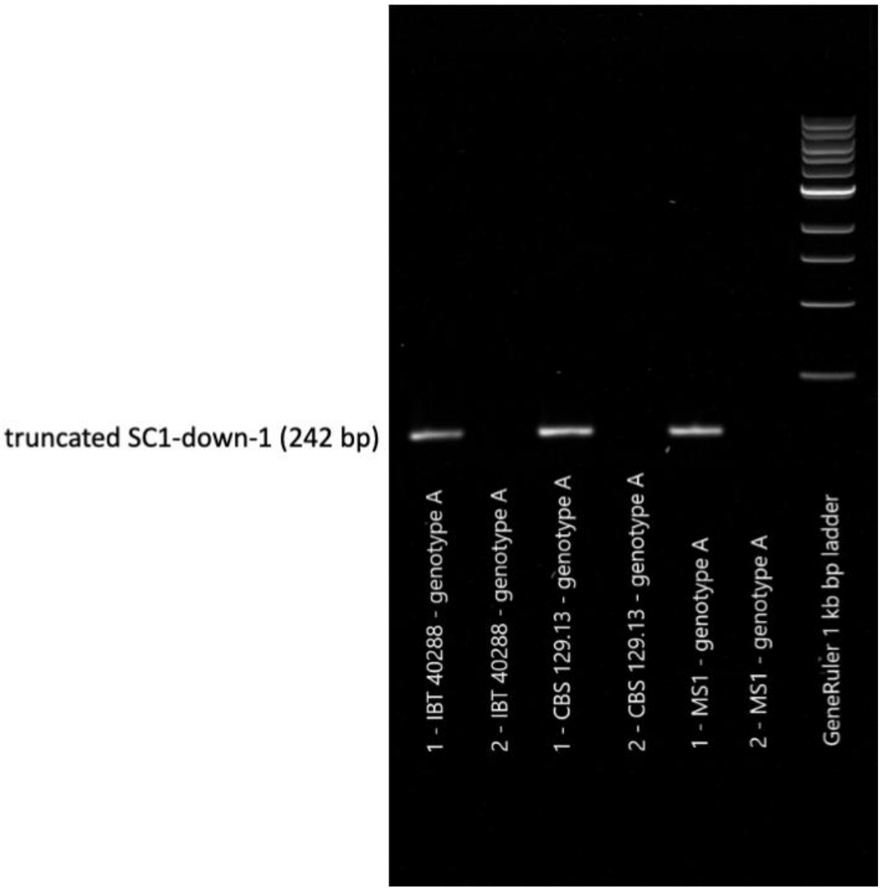

(C)

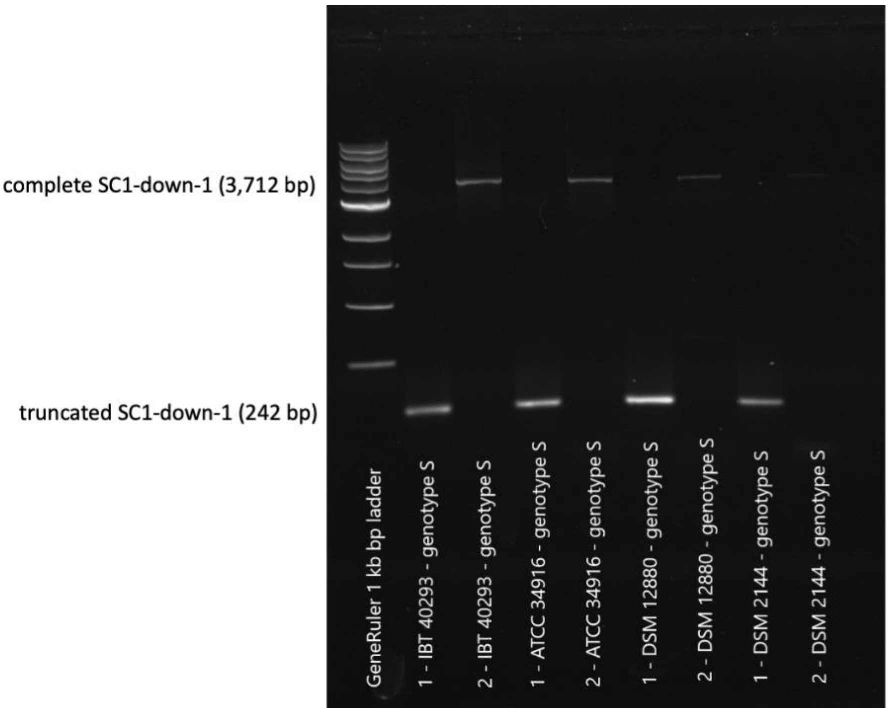

Figure S4. (A): Truncated SC1-down-1 gene in scaffold 1204 of IBT 40288 and A-type specific sequence in place of SC1 of S-type strains; (B) and (C): PCR for the presence of a complete and/or truncated SC1-down-1 gene sequence of *Stachybotrys chartarum* chemotype S and A strains. The position of the primers is shown in panel A. Primers: 1: A-gen1-F, A-gen1-R, 2: S-gen1-F, S-gen1-F. Lanes M:

GeneRuler 1,000 bp ladder; B: lane 1+2 *S. chartarum* IBT 40288 (A); lane 3+4 = *S. chartarum* CBS 129.13 (A); lane 5+6 = *S. chartarum* MS1 (A); B: lane 1+2 = *S. chartarum* IBT 40293 (S); lane 3+4 = *S. chartarum* ATCC 34916 (S); lane 5+6 = *S. chartarum* DSM 12880 (S); lane 7+8 = *S. chartarum* DSM 2144 (S). Expected fragment lengths: Complete SC1-down-1 3,712 bp, truncated SC1-down-1 242 bp (GCGGGCACCAGGTGGGCAAGCCGGCGAGTACTGGCATGGAAAAGCTCTTTGGCGACCTGA
TCCGCGTCAGCAAGGAGACGATCAATTTTGTGGACATCAAAAAGCTGGTGTCTCAGGACAA
TAACAAGTCGTGGCCGTCGGCCAGGGCCGTTGCTGATCGCGCTCACCATTTGATTTCAGAGC
CGGGAGAGTCCAAAACTCGAAAGAAGCATCACATAGCTCCACAGACCGGATGGACTTGA).

[1]CCAGGCGCAAGTGCTGATGGCAGGCGGCCAGCTCGATCATGCGCACCGTGGGGACCTCGA
GGAGGAAGTGGGAAAGGCGAAAAAGGAGGTAGAGGATAAATGGGACCACGAGATGCGTA
TTCCGCAACATGTCACAGGGCGAATGGAAGATGAAGAGGGATGAAATGAGCTGTTGCAAA
GATACTTGATTTGATAGTGATGGAATGGATGGCTAAGCAGTTGAATATTAATTATACTTAG[1][2]
TTATTTTAAGAAGTAAACTTTTTTAAATACCCCTTATATTAATTAAAGTATTTTAAATTGTTTT
AAATTATTTAAAAAGAAGTATTAAATATATAGAATTTAGAATTTAGTAATTATATTTATAAA
AATGTATTATATTATTTAGATTATTAAAGAAAAACTTAATAACTATAGAAT[2][3]GTATTATATA
AGTAGCTTAAGAGAAAATAGTATATTATAGATAAGGAATTAATAGAGACATACTACGTTTT
TTATAGGGACGCATAATATAATCCTTAGGGACACATAACTGACTACACCTCCGATTGGTCA
GAAAACAATCTGCCCTGCTATTCCCCGGCCCACCATATCGCAACCAGATGCCCTAACAGAG
CCATAGCTACAACGGCTACAGCAGTAGGCGCTAACAGCCTCGATAGCTTCAATCGGGCTGC
GGGTACCGCTATGGGCGCTATAGCTATTATTAAACCGGAGGCATCCTGCACAAGGCACAGT
ATGACAATATTACGCAGCTCAAATAACCAGAATAAGCCTCGTTGGTAGAGTCTAAGGGTCA
TGTTACCATACCACTCCCAATTAGGCATAGTGCAAGTAGTTTCCCTCAAATGGTCCCAGAAT
TGAGGGGCCAAGAGGGAATAGACACTAACAACAACATGACGGTCAAACAGAAATGGTCT
GTTTCGGCCTGGTACCACTCGGCAATGTGACTATAGAGCCGACCAGGGTGTTGGTGTAATC
AACTTCGCCAAACACGTACGAACCGCTGTTAGCCTTGAGCTCGATA[3]

Figure S5. Sequence connecting scaffold 1 (accession KL652499) and 1543 (accession KL651645) of IBT 40293; Forward Primer: F-crossing-1; Reverse Primer: R-crossing-1; [2]newly sequenced gap region; [1]scaffold 1; [3]scaffold 1543.

Table S2: Identity of trichodiensynthase- and satratoxin genes of *S. chartarum* (IBT 7711) with *Monosporascus cannonballus*

No.	Gene locus in *Monosporascus cannonballus* (CBS 609.92)	Cover (%)	Identity (%)	Scaffolds in *S. chartarum* (IBT 7711)	Similar trichodiensynthase genes
5	DL762_005686			n.f.	
6	DL762_005687	96	82.89	1385*	*tri*5***
7	DL762_005688	97	79.54	1385*	*tri*11 - cytochrome P450 monooxygenase****
8	DL762_005689	83	76.48	1385*	*tri*17/18****
9	DL762_005690	89	75.34	1285**	*tri*18/17****
10	DL762_005691	89	77.34	1385*	*tri*6 - biosynthesis transcription factor*****
11	DL762_005692	93	82.25	1385*	*tri*10 - putative regulatory protein****
12	DL762_005693	89	76.02	1385*	*tri*3 - 15-O-acetyltransferase****
13	DL762_005694	90	79.17	1385*	*tri*4 - cytochrome P450*****
14	DL762_005695	90	80.25	1385*	*tri*14***
15	DL762_005696			n.f.	*tri*12 - trichothecene efflux pump****
16	DL762_005697			n.f.	

n.f.: No significant similarity found on NCBI; *: CTC and SC3 containing scaffold; **: SC2 containing scaffold; ****S. chartarum*; *****Trichoderma* spp.; ******Myrothecium roridum.*
